# Inhibition of *NFE2L1* Enables the Tumor‐Associated Macrophage Polarization and Enhances Anti‐PD1 Immunotherapy in Glioma

**DOI:** 10.1111/cns.70488

**Published:** 2025-07-17

**Authors:** Qun Zhang, Qiusi Tian, Rongzhen Deng, Keli Liu, Shiqun Chen, Shaofan Hu, Zhengwen Zhang, Hongzhao Lu, Yiguo Zhang

**Affiliations:** ^1^ The Laboratory of Cell Biochemistry and Topogenetic Regulation College of Bioengineering and Faculty of Medical Sciences, Chongqing University Chongqing China; ^2^ Department of Neurosurgery 3201 Hospital of Xi'an Jiaotong University Health Science Center Hanzhong China; ^3^ Laboratory of Neuroscience Institute of Cognitive Neuroscience and School of Pharmacy, University College London London UK; ^4^ School of Biological Science and Engineering Shaanxi University of Technology Hanzhong China; ^5^ School of Life and Health Sciences Fuyao University of Science and Technology Fuzhou Fujian China

**Keywords:** anti‐PD1, glioma, *NFE2L1*, tumor‐associated macrophages

## Abstract

**Background and Objectives:**

A pivotal role of cancer (e.g., glioma) microenvironment is primarily executed by tumor‐associated macrophages (TAMs) in facilitating cancer immune evasion and even resisting immunotherapies. However, the molecular base for governing such functionality of TAMs remains poorly understood. Thereby, we here explore the impact of such a key regulatory transcription factor NFE2L1 (also called Nrf1) on glioma‐relevant TAMs.

**Methods:**

A set of combining in vivo and in vitro experimental approaches, e.g., by utilizing CRISPR‐Cas9 and overexpression plasmids to modulate NFE2L1 expression, and the resulting phenotypic changes in TAMs were evaluated. Besides, immunofluorescence, RT‐qPCR and flow cytometry were conducted to assay the infiltration of various immune cells, such as CD8+ T cells and M1‐type macrophages, in the glioma microenvironment, as well as their therapeutic response to anti‐PD1 treatment.

**Results:**

Deficiency of NFE2L1 causes a unique phenotypic switch in the TAMs from its pro‐cancer M2‐type to another anti‐cancer M1‐type, thereby inhibiting malignant progression of glioma. Such NFE2L1‐deficiency leads to significantly increases of CD8+ T cells and M1 macrophages within tumor tissues of glioma and hence enhances its sensitivity to anti‐PD1 therapy. Further experimental evidence has provided revealing a synergistic efficacy triggered by combined therapy of CD38 inhibitor with PD1 antibodies, significantly inhibited tumor growth, compared to that of their monotherapy. The mechanistic study unraveled that NFE2L1 enables for directly binding to those ARE sites within the promoter regions of both CD38 and PD‐L1 genes in order to govern their transcriptional expression.

**Conclusions:**

The aberrant role of NFE2L1 in the malignant progression of glioma was discovered in this study. It is of crucial significance to emphasize the potential of NFE2L1 inhibition as a strategic approach to enhance the efficacy of immunotherapeutic intervention. Overall, this discovery holds a substantial promise for advancement of innovative combination therapies, potentially enhancing treatment outcomes for individuals afflicted with glioma.

## Graphical abstract

High‐expressed *NFE2L1* contributes to maintaining the M2 macrophage in glioma. Knockout of *NFE2L1/Nrf1* can reverse the M2 phenotype of TAMs to the M1 phenotype. In vivo tumor growth is significantly inhibited in *NFE2L1* ^*−/+*^‐deficient heterogeneous mice. The activity of *CD38* and PDL‐1 inhibitors is enhanced by *NFE2L1* targeting the ARE sites. The anti‐PD‐1 therapeutic efficacy is strikingly enhanced on *NFE2L1* ^*−/+*^ tumor‐bearing mice.

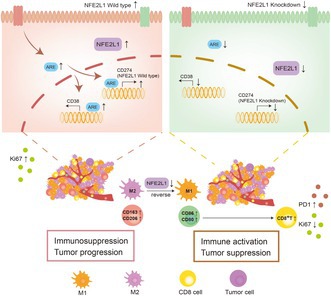

## 1 Introduction

Tumor‐associated macrophages (TAMs) are the predominant immune‐infiltrating cells in the tumor microenvironment. The polarization state of these cells directly influences tumor progression and the therapeutic response, making them attractive therapeutic targets [1, 2]. Studies have shown that TAMs exhibit high plasticity and are able to transition from the M2 phenotype to the M1 phenotype under specific conditions [3]. Importantly, the shift of macrophages from the M1 type, which suppresses tumors, to the M2 type, which promotes tumors, is a key feature of malignant cancer progression [4, 5, 6]. Therefore, targeting TAMs may be an effective strategy for treating glioma. However, whether their phenotypic changes are guided by specific microenvironmental cues remains largely unclear.

Nrf1 (nuclear factor erythroid 2‐related factor 1, encoded by *NFE2L1*) is a key transcription factor that regulates cellular stress response processes and plays a particularly important role in maintaining mitochondrial homeostasis [7], the cellular metabolic balance [8], and the redox balance [9]. Increasing evidence indicates that *NFE2L1* plays a crucial role in immune responses, particularly in inflammatory diseases [10, 11, 12]. Studies have shown that *NFE2L1* can regulate the homeostasis of mitochondrial proteins in mouse macrophages, thereby promoting dynamic changes in inflammatory responses [10]. Furthermore, *NFE2L1* can function by negatively regulating the M1 polarization and pro‐inflammatory responses of RAW264.7 macrophages and human monocytes [11].

Glioma is among the most prevalent primary tumors of the central nervous system (CNS) [13]. Despite considerable advancements in multimodal diagnosis and treatment, the highly invasive characteristics of glioma and the intricacies of the tumor microenvironment (TME) result in exceedingly poor clinical outcomes for patients [14, 15], necessitating groundbreaking therapeutic approaches. The extensive infiltration of TAMs underscores the complexity of the glioma microenvironment [16]. TAMs significantly influence various phases of tumor development, including progression, invasion, angiogenesis, metastasis, and the emergence of drug resistance [2, 3, 17, 18]. Consequently, clarifying the mechanisms of TAM infiltration in the glioma microenvironment is crucial for clinical treatment. CD38, as a multifunctional transmembrane glycoprotein, plays a crucial role in regulating immune responses and the TME. Not only does CD38 function as a NAD + ‐hydrolyzing enzyme, but it also modulates intracellular calcium ion release, thereby influencing cellular metabolism and function [19]. In gliomas, CD38 expression is closely associated with tumor invasiveness and patient prognosis. Studies indicate that high CD38 expression correlates with an immunosuppressive TME, potentially facilitating tumor immune evasion by promoting the recruitment of regulatory T cells (Tregs) and myeloid‐derived suppressor cells (MDSCs) [20, 21].

In the present investigation, we explored the regulatory role of *NFE2L1* in the phenotypic polarization of TAMs and the proliferation of glioma cells. Our findings indicate that the downregulation of *NFE2L1* facilitates a transition of TAMs from the M2 phenotype to the M1 phenotype, which in turn hinders the aggressive proliferation of glioma. Importantly, our study shows that inhibiting *NFE2L1* boosts anti‐PD1 immunotherapy effectiveness in glioma, suggesting it as a valuable therapeutic target for enhancing TAMs and antitumor responses.

## 2 Materials and Methods

### 2.1 Ethics Statement

The glioma specimens were obtained from patients at 3201 Hospital in Hanzhong, China, from 2014 to 2024. The research protocol garnered approval from the ethics committee (TYYLKYJJ‐2022‐014, as outlined in the Supporting Information), and informed consent was secured from all participating patients. The patients' clinical information is shown in Table S1.

### 2.2 Animals

Due to the embryonic lethality observed in *NFE2L1* knockout (*NFE2L1* ^−/−^) mice [22], this investigation chose to utilize *NFE2L1* heterozygous (*NFE2L1* ^−/+^) mice for the experimental procedures (refer to Table S2). The *NFE2L1* ^−/+^ mice were sourced from Cyagen Biosciences Inc. The animal experiments for the study were approved by the Animal Management Committee of Chongqing University and followed ethical standards.

Four‐week‐old C57BL/6 mice were anesthetized using isoflurane, implanted with 10 × 10^4^ GL261 cells expressing luciferase to establish an intracranial glioma model, euthanized 3 weeks later, and tumor growth was assessed by bioluminescence imaging. Mouse brains were fixed in PFA before sectioning. In the glioma model, GL261 cells were implanted in *NFE2L1*−/+ C57BL/6 mice across four treatment groups: control+lgG, CD38 inhibitor, anti‐PD‐1, and combination, with five mice each. The CD38 inhibitor was given at 25 mg/kg every 2 days and anti‐PD‐1 at 2.5 mg/kg every 4 days. Tumor tissues were collected after 21 days for analysis.

### 2.3 Bioinformatics Analysis

*NFE2L1*'s correlation with immune cells was assessed using immuneeconv, while ggpubr and ggExtra analyzed its association with tumor immune cell infiltration. Spearman analysis of *NFE2L1* and macrophage markers was conducted via GEPIA, supplemented by Sangerbox, Clinical Bioinformatics Assistant, and TIMER.

### 2.4 Cell Culture and Treatment

The U251, LN229, and THP‐1 cell lines were obtained from the Chinese Academy of Sciences Cell Bank. THP‐1 was grown in Pricella medium, while U251 and LN229 were maintained in DMEM high‐glucose medium under controlled conditions.

To generate *NFE2L1*‐deficient THP‐1 cells, we employed sgRNA‐targeting knockout plasmids preserved in our laboratory [23]. Cultures at 70%–80% confluence had plasmids transfected with Lipofectamine 3000, medium changed after 8 h, and *NFE2L1*‐deficient cell lines isolated after 24 h.

After differentiation of THP‐1 cells into a macrophage‐like state, 2 μg of *NFE2L1* overexpression plasmid (pCDNA3.1‐*NFE2L1*) or empty vector control was transfected using Lipofectamine 3000. The *NFE2L1* overexpression plasmid was constructed and stored in our laboratory. Cells were collected 48 h after transfection, and the levels of Nrf1 protein and mRNA were verified by western blot and qPCR, respectively. The experiment was repeated three times, and the data are presented as mean ± SD.

M2 macrophages were derived from THP‐1 cells stimulated with PMA for 24 h to form M0 macrophages, which were then treated with IL‐4 and IL‐13 for 48 h. TAMs were generated by coculturing THP‐1 cells with supernatants from U251 and LN229 cells, followed by collection for analysis.

### 2.5 Real‐Time qPCR Analysis

Total RNA was isolated with RNAex Pro Reagent, followed by cDNA synthesis using a reverse transcription kit for verification, and RT–qPCR was conducted per established protocols [7]. β‐actin served as the reference gene, and the outcomes were represented as 2^−△△CT^. The primers utilized and their sequences are displayed in Table S3.

### 2.6 Western Blotting Analysis

After protein extraction, concentrations were measured with a BCA kit, separated by SDS‐PAGE, and transferred to a PVDF membrane. Membranes were blocked for 1 h incubated overnight with primary antibody, and then with secondary antibody for 1 h for protein visualization using the ECL kit. Antibody details are in Table S4.

### 2.7 Immunofluorescence

Immunofluorescence of cells or tumors involved fixing, permeabilizing, and blocking samples, followed by overnight primary antibody incubation and secondary antibody staining. Nuclear staining and imaging were done using a digital slide scanner, with antibody details in Table S4.

### 2.8 Immunohistochemistry and HE Staining

For immunohistochemistry, 5 μm sections were deparaffinized, rehydrated, and treated with EDTA, hydrogen peroxide, and antibodies, followed by DAB chromogen. For H&E staining, mouse brain tissues were fixed, embedded, sectioned at 10 μm, and stained with hematoxylin and eosin, with images captured using a digital slide scanner.

### 2.9 Transwell Assay

U251 or LN229 cells were seeded in the transwell upper compartment and macrophages in the lower chamber. After 48 h, the upper chamber was removed, and cells were fixed, stained with crystal violet, and counted under a microscope to assess invasion ability.

### 2.10 Wound Healing Assay

Wound healing assay was done on U251 or LN229 cells at 90% confluence, scratching the surface with a pipette tip, washing with PBS, and photographing after 24 h with macrophage conditioned medium.

### 2.11 RNA Sequencing for Transcriptome Analysis

RNA was isolated with TRIzol, mRNA reverse transcribed to cDNA, purified, and a library developed; raw sequencing data were analyzed on OmicShare.

### 2.12 Flow Cytometry

TAMs were isolated, incubated with anti‐CD163 and anti‐CD206 antibodies at 4°C for 30 min, washed twice, and resuspended in 200 μL buffer. Analysis was performed using a FACS Calibur flow cytometer and data processed with FlowJo software; antibody details are in Table S4.

### 2.13 Mass Spectrometry

Glioma tissues from *NFE2L1* ^−/+^ and WT C57BL/6 mice were analyzed using CyTOF by Puluting Health Technology in Hangzhou. Tumor tissues were digested with a Miltenyi kit, filtered, and stained with metal‐labeled antibodies and DNA (193Ir). Cells were also incubated with antibodies for intracellular molecules. Data were normalized in FlowJo, and CD45+ cells underwent X‐shift clustering analysis in R to identify immune subpopulations. Antibody details are in Table S5.

### 2.14 Dual Luciferase Activity Assay

ARE sequences from PD‐L1 or CD38 promoters were inserted into pGL3 as ARE‐Luc reporters. When 293 T cells reached 80% density, PD‐L1 or CD38 ARE‐Luc vectors, pRL‐TK (Renilla), and *NFE2L1* expression vector were co‐transfected. After 24 h in complete medium with 10% FBS, luciferase activity was measured using the Dual‐Luciferase Reporter Gene Detection Kit II.

### 2.15 Statistical Analysis

Statistical analyses used GraphPad Prism 9.5.1, showing data as mean ± SD. Group comparisons employed independent *t*‐test, with *p* < 0.05 as significant.

## 3 Results

### 3.1 NFE2L1 is Significantly Upregulated in Glioma Patients and is Closely Related to Immune Infiltration

**FIGURE 1:**
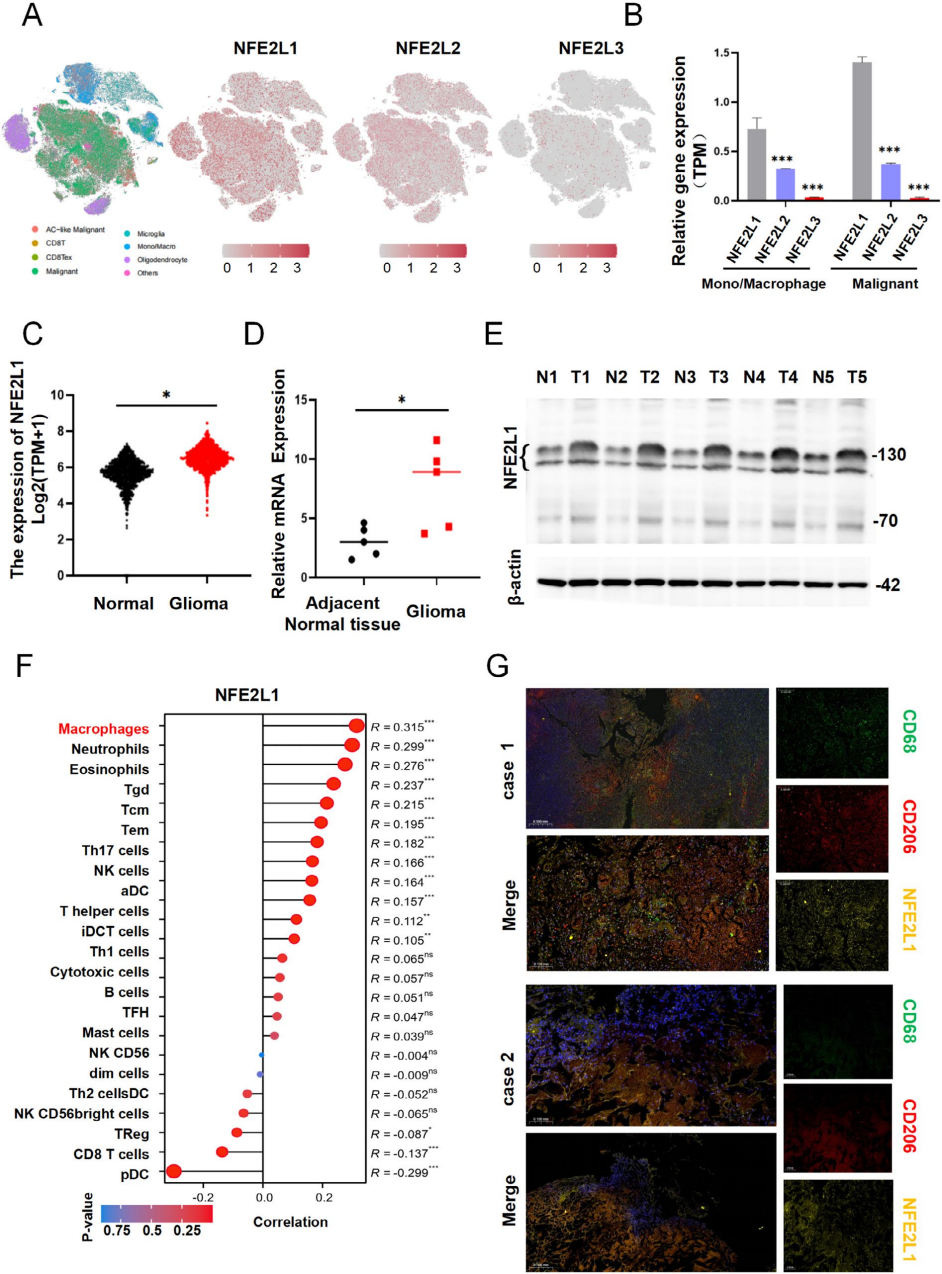
NFE2L was significantly upregulated in the tumors of glioma patients and was closely related to the level of immune infiltration. (A) Nuclear factor erythrocyte 2‐like (Nfe2l) gene family expression levels in glioma cells and the infiltration of immune cells. Unpaired Student's *t*‐test. Error bar = SD. (B) *NFE2L1* was expressed mainly in Mono/Macrophages and glioma cells (malignant). (C) *NFE2L1* expression in human normal brain tissues and glioma samples from the TCGA database. (D) The mRNA level of *NFE2L1* was increased in human glioma tissue samples, as determined via RT–qPCR. (E) The protein level of *NFE2L1* was increased in human glioma tissue samples, as determined by Western blot analysis. (F) *NFE2L1* was most closely related to the infiltration of macrophages among the infiltrating immune cells in glioma. (G) *NFE2L1* colocalized with CD163 and CD206 in macrophages. The experiments were performed in triplicate, and the data are presented as the means ± SDs, *p < 0.05, ***p < 0.001 versus the control group.

To investigate the role of *NFE2L1* in the glioma microenvironment, we initially established a single‐cell transcriptional atlas derived from tumors of glioma patients, focusing on the expression profiles of the nuclear factor erythrocyte 2‐like (Nfe2l) gene family. Our findings indicated that *NFE2L1* is predominantly expressed in glioma tumor tissues, with NFE2L2 exhibiting lower expression levels, whereas NFE2L3 was expressed at minimal levels (Figure 1A). Notably, the expression of *NFE2L1* was significantly elevated in malignant glioma cells and Mono/Macrophages compared with that of NFE2L2 and NFE2L3 (Figure 1B). This observation implies that *NFE2L1* may play a crucial role in modulating tumor cell proliferation and the infiltration of immune cells.

We focused on elucidating the function of *NFE2L1* within the context of glioma. Our initial analysis revealed that the mRNA expression of *NFE2L1* was significantly greater in tumor samples from patients than in normal brain tissue (Figure 1C). Validation through RT–qPCR and immunoblotting further confirmed the upregulation of *NFE2L1* in glioma tumors relative to normal brain tissue (Figure 1D,E). Further investigations revealed that among the immune‐infiltrating cell types in glioma, *NFE2L1* displayed the strongest association with macrophage infiltration (Figure 1F). Immunofluorescence assays revealed the colocalization of *NFE2L1* with CD163 and CD206 in macrophages (Figure 1G). Collectively, these findings underscore the pivotal role of *NFE2L1* in the context of TAMs.

### 3.2 NFE2L1 Is Essential for the Generation of TAMs With the M2 Phenotype

**FIGURE 2:**
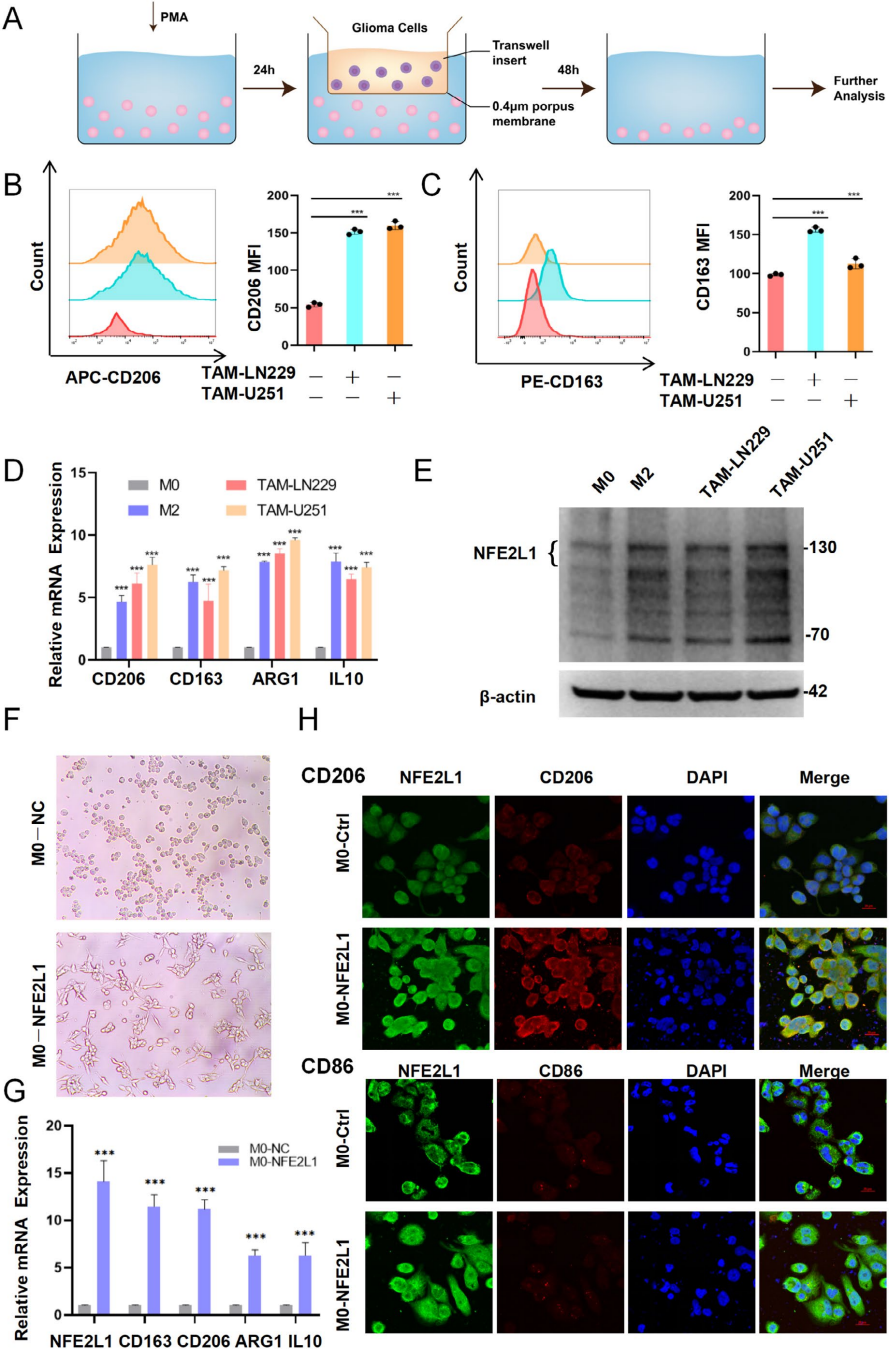
*NFE2L1* is upregulated in M2‐like TAMs, and its high expression promotes the M2 polarization of macrophages. (A) Experimental diagram of the generation of TAMs. (B) Flow cytometry analysis revealed that the expression of CD206 and (C) CD163 was significantly increased in TAMs cocultured with U251 or LN229 cells. (D) The relative expression of M2 markers (CD163, CD206, ARG1, and IL‐10) was significantly increased in M2 macrophages and TAMs cocultured with U251 or LN229 cells for 48 h. (E) The protein level of *NFE2L1* was significantly increased in M2 macrophages and TAMs, as determined via Western blot analysis. (F) Macrophages that overexpress *NFE2L1* exhibited typical M2 macrophage morphology. (G) The relative expression of M2 markers (CD163, CD206, ARG1, and IL‐10) was significantly increased in macrophages overexpressing *NFE2L1*. (H) *NFE2L1* colocalizes with the M2 marker CD206 in macrophages but does not colocalize with the M1 marker CD86. The experiments were performed in triplicate, and the data are presented as the means ± SDs; ***p < 0.001 vs. the control group.

Based on these results, *NFE2L1* may play a significant role in the TAMs of glioma. To further investigate this, we developed an in vitro model of TAM induction, as illustrated in Figure 2A. Flow cytometry revealed that the induced TAMs highly expressed CD206 (Figure 2B) and CD163 (Figure 2C), which are markers of M2‐type TAMs. Concurrently, RT–qPCR analysis also revealed that the expression levels of the M2‐type markers CD163, CD206, ARG1, and IL10 in TAMs were significantly increased (Figure 2D). These findings indicate that the TAMs we induced in vitro exhibit the phenotype of M2‐type macrophages. Subsequent detection revealed that the expression levels of *NFE2L1* were significantly increased in M2‐type macrophages and TAMs (Figure 2E).

To further validate the role of *NFE2L1* in the polarization process of macrophages, we overexpressed *NFE2L1* in macrophages, and the results revealed that the macrophages exhibited significant invasive branching (Figure 2F). Concurrently, after overexpressing *NFE2L1*, the expression levels of the M2 markers CD163, CD206, ARG1, and IL10 were significantly increased (Figure 2G). Therefore, we further conducted immunofluorescence experiments, and the results revealed that in macrophages, *NFE2L1* could be colocalized with CD206 (Figure 2H), whereas *NFE2L1* was not colocalized with CD86.

### 3.3 NFE2L1 ^−/−^ Macrophages Inhibit Glioma Cell Migration

**FIGURE 3:**
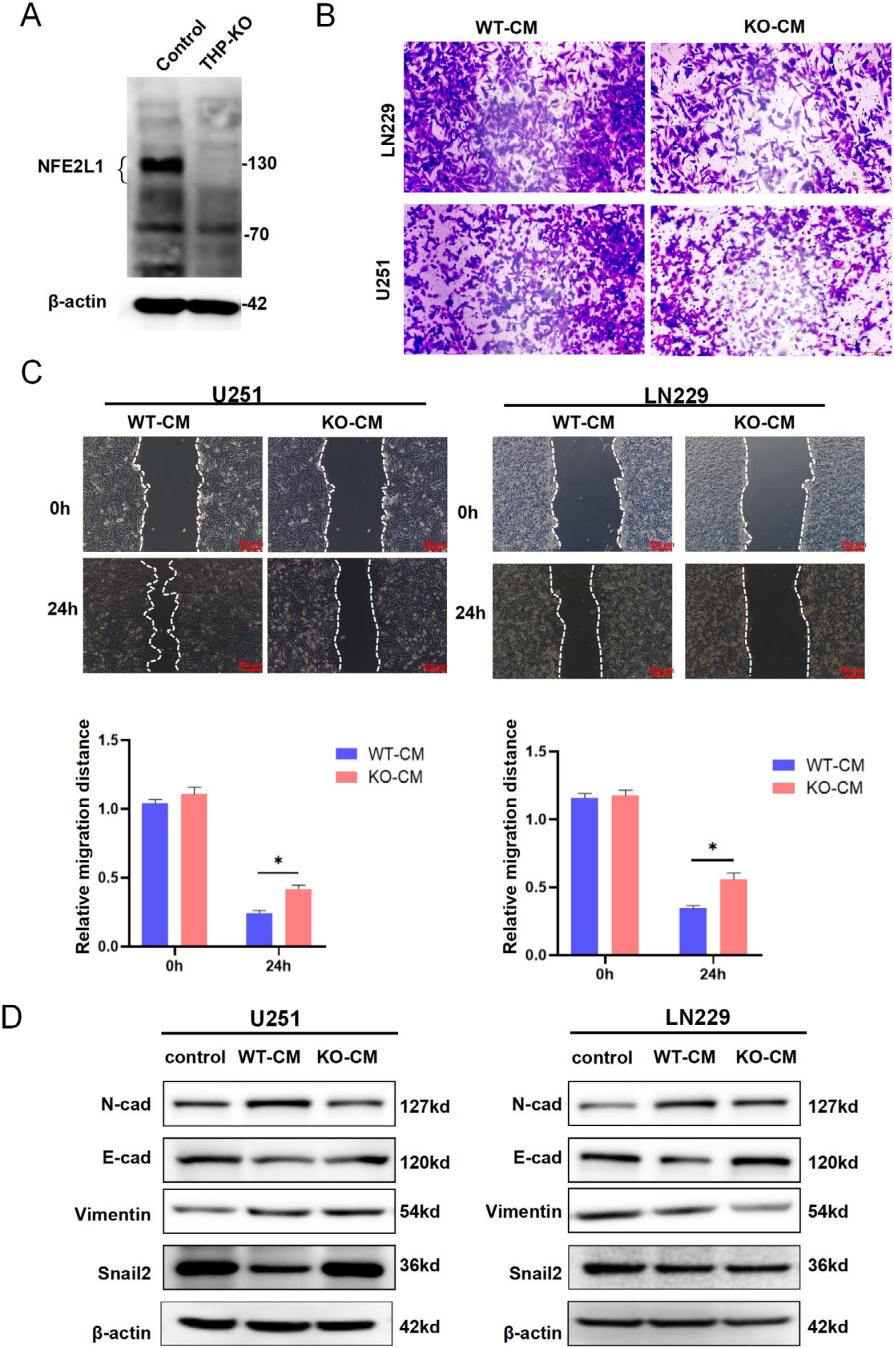
*NFE2L1* knockout in macrophages inhibits the migration of glioma cells. (A) Western blot analysis confirmed the successful establishment of the *NFE2L1* knockout THP‐1 cell line. (B) Macrophages with *NFE2L1* knockout inhibited the invasion of U251 and LN229 cells, as measured by Transwell assays. (C) Macrophages with *NFE2L1* knockout suppress the migration of U251 and LN229 cells, as measured by wound healing assays. (D) Macrophages with *NFE2L1* knockout regulated the expression of EMT markers in U251 and LN229 cells. The experiments were performed in triplicate, and the data are presented as the means ± SDs; *p < 0.05 vs. the control group.

To delve deeper into the potential impact of *NFE2L1* expression in macrophages on the functionality of glioma cells, we engineered THP‐1 cells with a targeted knockout of *NFE2L1*. Western blot analysis confirmed the successful ablation of *NFE2L1* in these cells (Figure 3A). Transwell assays revealed that CM from *NFE2L1*‐deficient macrophages significantly inhibited the invasive ability of LN229 and U251 cells (Figure 3B). Furthermore, wound healing assays demonstrated that CM from *NFE2L1* knockout macrophages markedly inhibited the migratory behavior of U251 and LN229 cells (Figure 3C). Given the pivotal role of epithelial–mesenchymal transition (EMT) in the invasion and metastasis of glioma [24], we treated LN229 and U251 cells with CM from *NFE2L1*‐deficient macrophages. This treatment led to a notable increase in the expression of the EMT marker E‐cadherin, while the expression levels of N‐cadherin, vimentin, and Snail2 were substantially reduced. Interestingly, Snail2 exhibited an inverse expression pattern in U251 cells (Figure 3D). The potential reasons for the differential expression of Snail2 in U251 and LN229 KO‐CM may be attributed to cell line‐specific signaling pathway backgrounds; U251 and LN229 cells have distinct genetic and epigenetic characteristics. U251 may exhibit higher baseline expression of pro‐EMT factors (e.g., TGF‐β1 and WNT/β‐catenin). KO‐CM treatment could further activate Snail2 transcription by relieving certain inhibitory regulation (e.g., reduced miR‐34a or GSK3β activity). In contrast, LN229 cells may rely on other EMT regulators (e.g., ZEB1 or Twist1), leading to the downregulation of Snail2 in KO‐CM due to compensatory pathway suppression. This still requires further experimental validation [25, 26, 27, 28].

### 3.4 NFE2L1 Deficiency Suppresses Gliomagenesis

**FIGURE 4:**
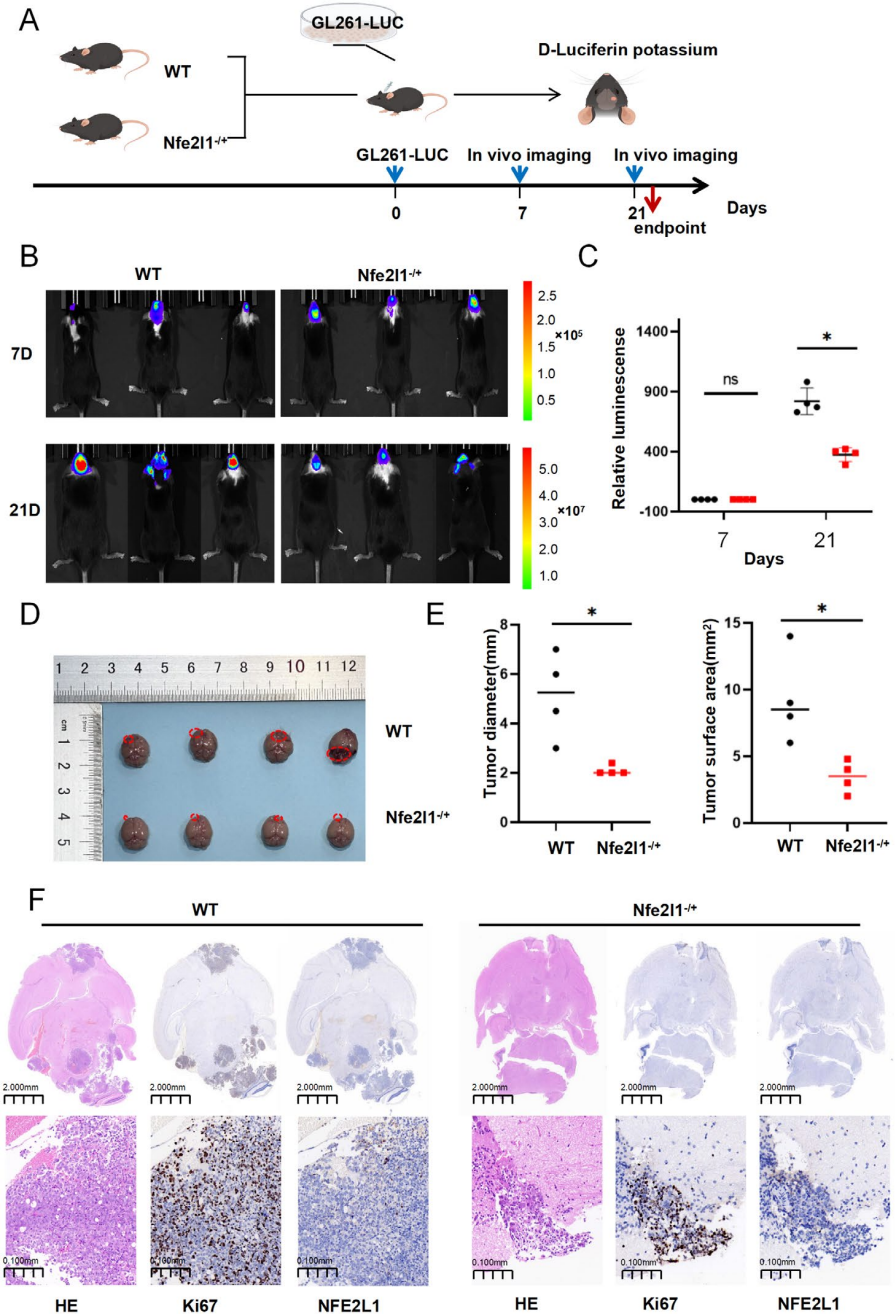
The absence of *NFE2L1* inhibits the growth of glioma in vivo. (A) Experimental design for constructing an orthotopic glioma mouse model. (B) GL261‐luc cells were injected into the brains of wild‐type C57BL/6 mice and *NFE2L1* ^−/+^ C57BL/6 mice. (C) Bioluminescence imaging analysis of tumor growth was performed at 7 and 21 days postinjection, and quantification was performed (mean ± SD; *n* = 4). (D) The effect of *NFE2L1* knockout on tumor growth was observed, and the tumor diameter and surface area were quantitatively analyzed (mean ± SD; *n* = 4) (E). (F) Representative images of HE staining and IHC staining (Ki67 and *NFE2L1*). (Five mice were assigned to each group, and data from four mice are shown because one mouse died before reaching the experimental endpoint.). *p < 0.05 versus the control group.

Acknowledging the potential role of *NFE2L1* in the malignant progression of glioma, we hypothesized that *NFE2L1* could facilitate glioma development in vivo. To evaluate this hypothesis, we employed the GL261 glioma cell line, which is engineered to stably express the luciferase reporter gene. We subsequently implanted GL261‐Luc cells into the brains of both wild‐type C57BL/6 mice and *NFE2L1* heterozygous C57BL/6 mice (Figure 4A). In vivo bioluminescence imaging indicated a significant reduction in luminescent signals in the *NFE2L1* heterozygous group compared to the control group at 21 days post‐implantation (Figure 4B,C). Furthermore, the measurements of tumor diameter and surface area were substantially lower in the *NFE2L1* heterozygous group than those observed in the control group (Figure 4D,E). Histological analysis through hematoxylin and eosin (HE) staining, accompanied by Ki67 proliferation assays, revealed that tumor growth and cellular proliferation were markedly inhibited in C57BL/6 mice with *NFE2L1* deficiency following the implantation of GL261 glioma cells (Figure 4F).

### 3.5 Inhibition of NFE2L1 Reversed the M2 Phenotype to the M1 Phenotype in TAMs From Glioma

**FIGURE 5:**
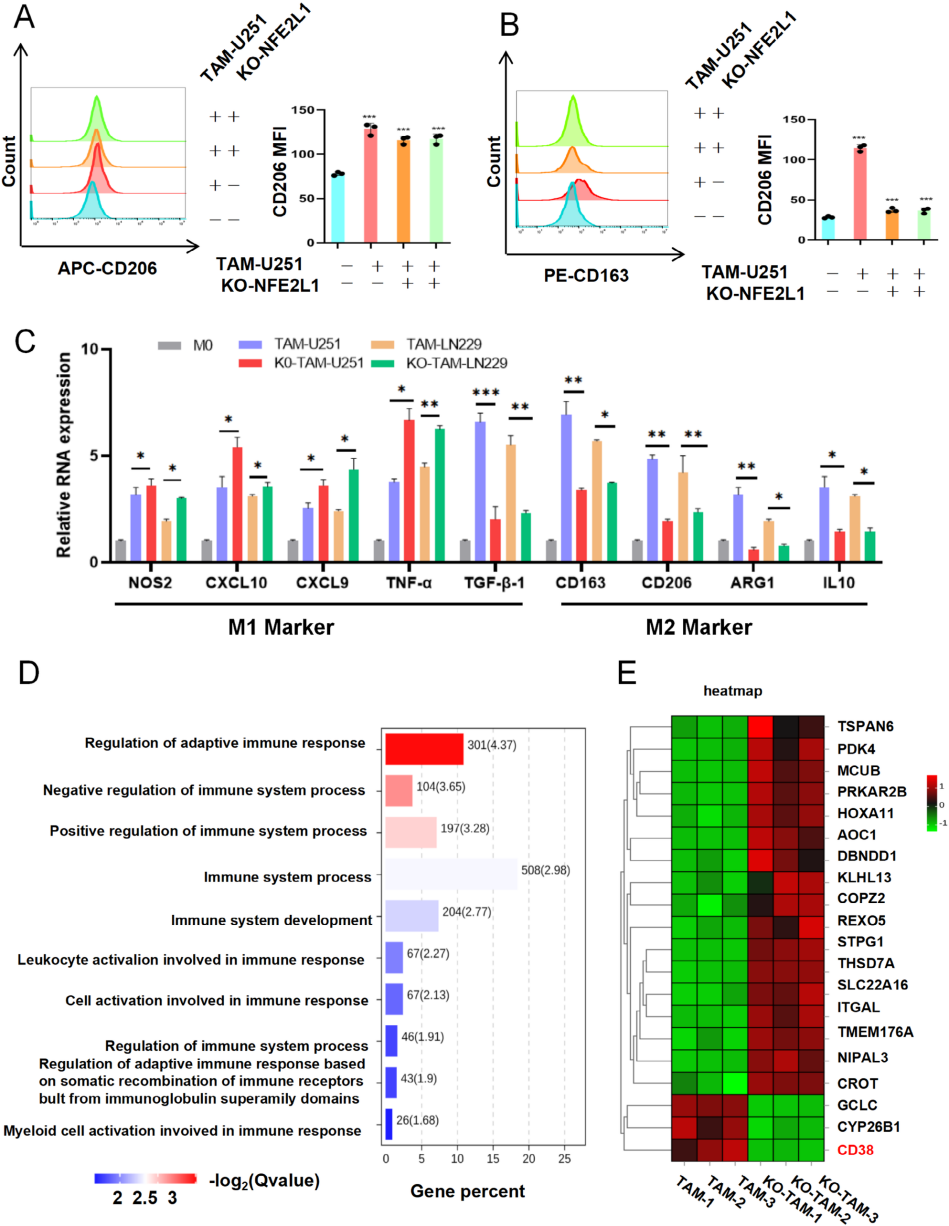
Knockout of *NFE2L1* reversed the M2 phenotype to the M1 phenotype in glioma tumor‐associated macrophages. (A, B) Flow cytometry analysis revealed that loss of *NFE2L1* resulted in significantly reduced levels of the M2 markers CD206 and CD163 in TAMs. (C) The absence of *NFE2L1* inhibited the relative expression of M2 markers in TAMs cocultured with U251 or LN229 cells but promoted the relative expression of M1 markers. (D) GO enrichment was used to characterize the immune signature pathway. (E) Heatmap of the top 20 differentially expressed genes. The experiments were performed in triplicate, and the data are presented as the means ± SDs, *p < 0.05, **p < 0.01, ***p < 0.001 versus the control group.

Is *NFE2L1* involved in regulating the transition between the M1 and M2 phenotypes in TAMs? We stimulated TAMs with the supernatant of glioma cells. Flow cytometry analysis revealed that loss of *NFE2L1* resulted in significantly reduced levels of the M2 markers CD206 and CD163 in TAMs (Figure 5A,B). Specifically, macrophages were stained using anti‐CD206 and anti‐CD163 antibodies (Table S4 for details). FACS Calibur flow cytometer was used for analysis, and FlowJo software was used to process the data. Importantly, the RT–qPCR results revealed that after the deletion of *NFE2L1*, the expression levels of the M1 TAM markers NOS2, CXCL10, CXCL9, and TNF‐α were significantly increased. The expression of TGF‐β1, CD163, CD206, ARG1, and IL10 in M2‐type TAMs was significantly inhibited (Figure 5C).

Further enrichment analysis of these DEGs revealed a substantial correlation between *NFE2L1* expression and immune‐related features, including the regulation of adaptive immune responses, leukocyte activation involved in immune responses, and myeloid cell activation during immune processes (Figure 5D). Additionally, we observed a significant downregulation of the expression of CD38, a molecule integral to immune regulatory mechanisms, following the ablation of *NFE2L1* (Figure 5E).

### 3.6 NFE2L1 Deficiency Resulted in Immune Activation, as Determined via Mass Spectrometry Analysis, and Enhanced Sensitivity to Anti‐PD1 Therapy in Glioma

**FIGURE 6:**
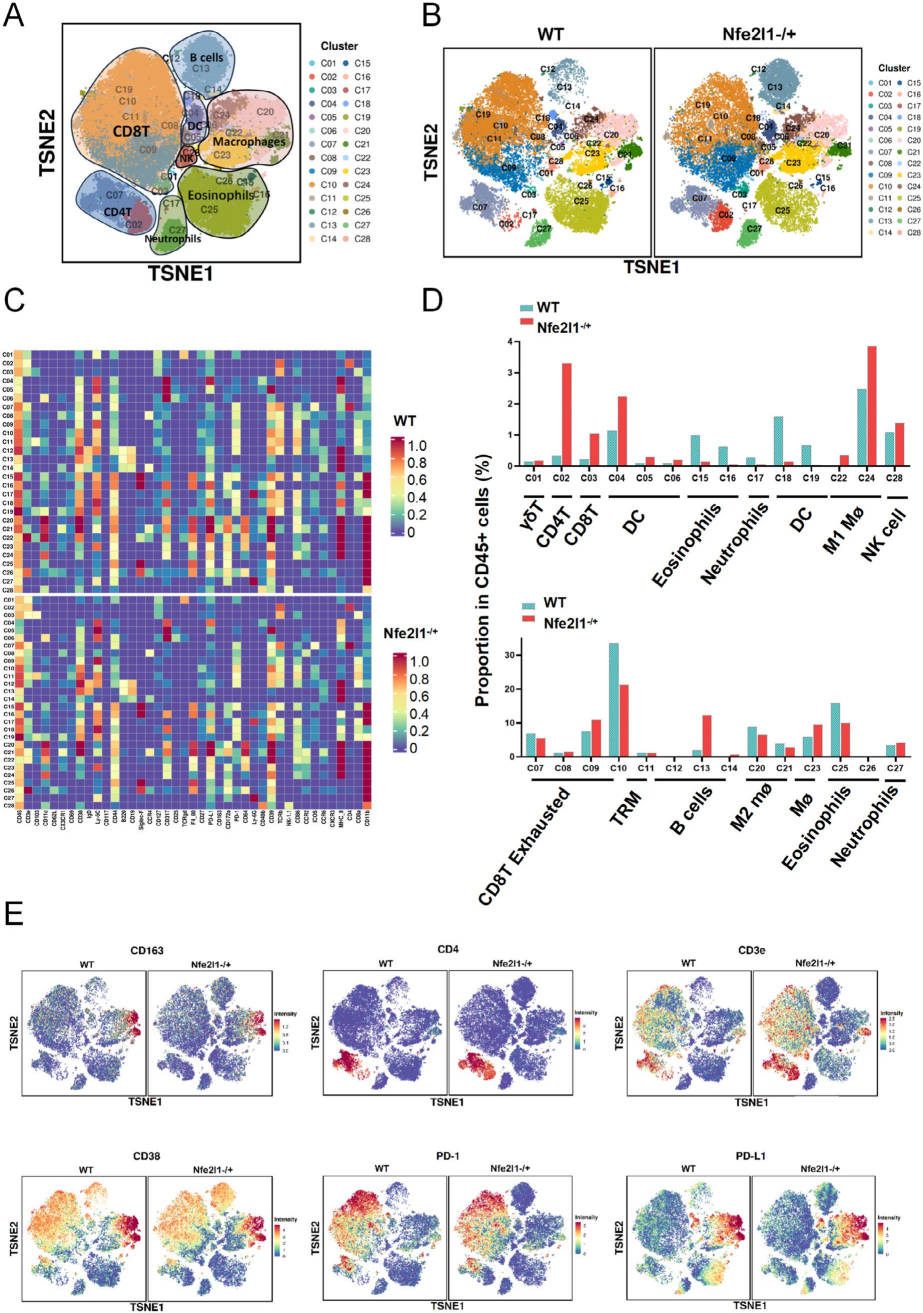
Based on mass spectrometry analysis, *NFE2L1* deficiency resulted in immune activation and increased sensitivity to anti‐PD1 therapy in glioma. (A, B) TSNE plot showing the distributions of 28 cell clusters in the respective samples. (C) A total of 28 cell clusters were divided, and we defined the respective cell clusters. (D) Histogram showing the number of the respective cell clusters in different groups. (E) TSNE plot showing the distributions of CD4, CD3e, CD163, CD38, PD1, and PD‐L1 expression in subcutaneous glioma tumors from different groups.

In our subsequent analysis, we sought to determine whether variations in *NFE2L1* expression are correlated with shifts in the immune microenvironment of glioma tumors. Mass spectrometry was employed to analyze samples derived from both WT and *NFE2L1* ^−/+^ tumor model mice. A total of 28 cellular clusters were delineated via distinct cellular markers, and the expression patterns of 41 markers across these clusters were mapped to elucidate cellular functional states (Figure 6A–C). In the absence of *NFE2L1*, we observed diminished infiltration of M2 macrophages and exhausted CD8+ T cells within the tumors, whereas the presence of CD8+ T cells and M1 macrophages tended to increase (Figure 6D). Strikingly, within the *NFE2L1*‐null cohort, there was a marked upregulation of the immune activation markers CD4 and CD3e (Figure 6E).

### 3.7 CD38 Inhibitors Enhanced the Anti‐PD‐1 Therapeutic Effect on NFE2L1 ^−/+^ Loaded Tumor Mice

Given that CD38 is recognized as a promising therapeutic target in glioma treatment [29], and that *NFE2L1* suppression heightened glioma responsiveness to anti‐PD1, we hypothesized that a synergistic approach involving anti‐PD1 monoclonal antibodies (mAbs) and CD38 inhibitors could potently suppress glioma progression.

**FIGURE 7:**
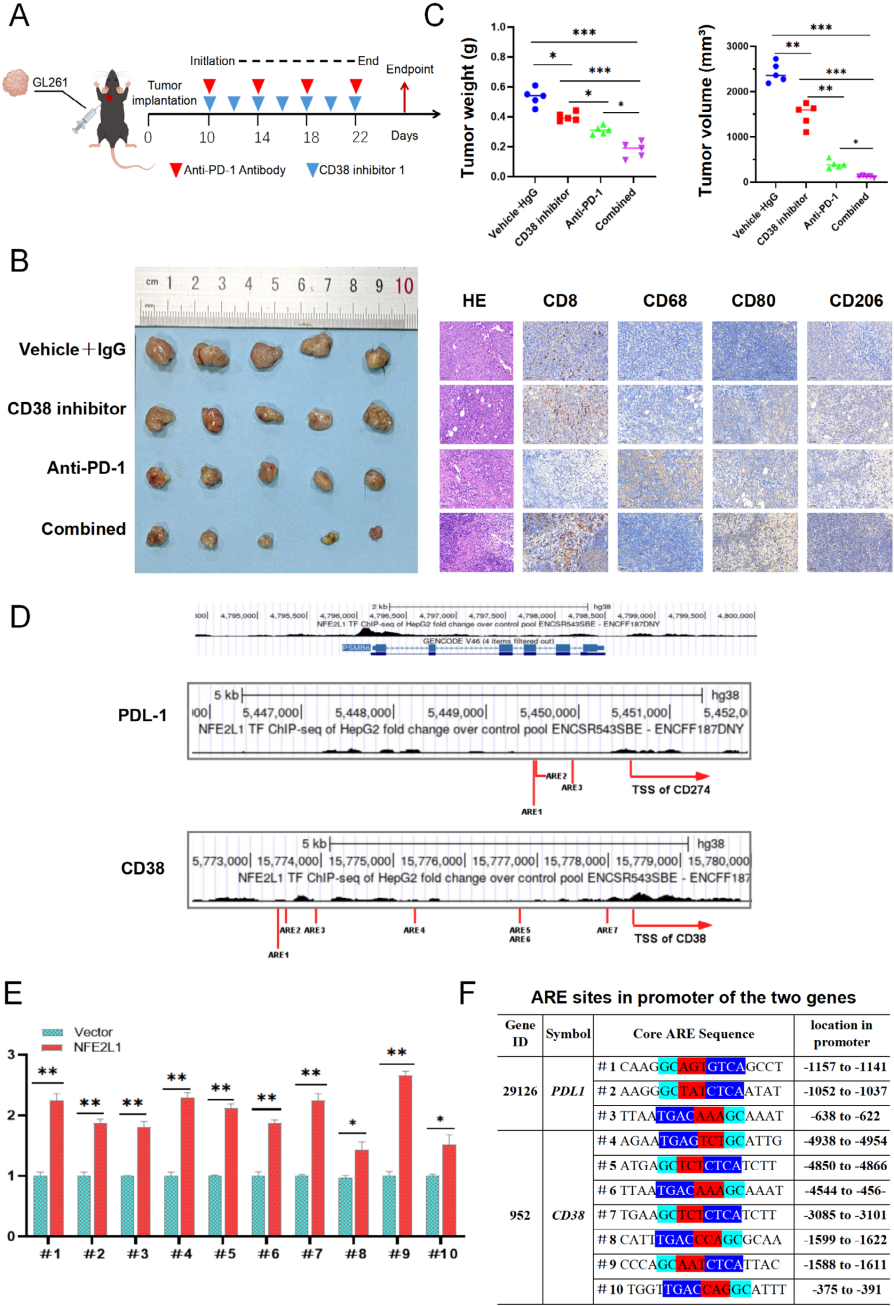
Blocking CD38 signaling enhances the efficacy of anti‐PD‐1 therapy in mice with *NFE2L1* knockdown. (A) Experimental design of anti‐PD‐1 and CD38 inhibitor therapy in *NFE2L1* knockout GL261 subcutaneous tumors in mice. (B) Images of subcutaneous tumors in each group and immunohistochemical staining images of CD8, CD68, CD80, and CD206. (C) The weight and volume statistics of the subcutaneous tumors in the different groups are shown (mean ± SD; *n* = 5). (D) ChIP‐sequencing data for *NFE2L1* (obtained from the Encode database) in the promoter regions of PDL‐1 and CD38. The transcription start sites and multiple ARE sites in the gene promoter regions are marked. (E) The relative luciferase activities of PDL1‐luc and CD38‐luc were determined in U251 cells coexpressed with each reporter gene and pRL‐TK (as an internal reference) plus an expression construct for the *NFE2L1* or empty pcDNA3 vector. (F) The consensus ARE sites within the promoters of PDL‐1 and CD38 are listed herein. The experiments were performed in triplicate, and the data are presented as the means ± SDs, *p < 0.05, **p < 0.01, ***p < 0.001 vs. the control group (five mice per group).

To validate this hypothesis, we subcutaneously implanted GL261 cells into WT and *NFE2L1* ^−/+^ C57BL/6 mice and subsequently administered various treatments (Vehicle + IgG group, CD38 inhibitor group, Anti‐PD‐1 group, and CD38 inhibitor + Anti‐PD‐1 group) to evaluate their tumor‐inhibiting potential (Figure 7A). The data indicated that both CD38 inhibition and anti‐PD‐1 therapy significantly inhibited tumor growth compared with that in the vehicle + IgG group, with the combination therapy exhibiting the most pronounced therapeutic benefits (Figure 7B,C).

### 3.8 NFE2L1 Targets Are Sites That Activate CD38 and PDL‐1

Recent investigations have demonstrated that *NFE2L1* plays a pivotal role in regulating gene expression by directly interacting with the consensus AU‐rich element (ARE) located within the regulatory regions of target genes [7]. The ARE sequence (5′‐TGAC/GnnnGC‐3′), recognized as a prominent cis‐acting enhancer element [30], led us to hypothesize that *NFE2L1* may influence the expression of CD38 and PD‐L1 through the activation of ARE sites. Supporting this hypothesis, ChIP‐seq data obtained from the ENCODE database unveiled several peak signals indicative of *NFE2L1*'s active binding to multiple ARE sites found within the promoter regions of PD‐L1 and CD38 (Figure 7D). Furthermore, experimental results indicated that the presence of *NFE2L1* could markedly enhance the activity of a luciferase reporter gene governed by the ARE site (Figure 7E). The ARE sequences identified within the promoter regions of PD‐L1 and CD38 were individually subcloned and introduced into the pGL3‐Promoter vector, thereby generating the corresponding ARE‐Luc reporter genes (Figure 7F).

## 4 Discussion

*NFE2L1* interacts with small Maf (sMAF) heterodimers to associate with antioxidant response elements (AREs), leading to the activation of specific target genes [31, 32]. As a key transcription factor, *NFE2L1* is integral to numerous physiological processes, which encompass redox signaling, cellular metabolism, and the regulation of the proteasome [31, 33]. This factor is closely linked to the pathogenesis of several diseases, including cardiovascular conditions [34], inflammatory diseases [35, 36], and autoimmune diseases [37, 38, 39]. Glioma, recognized as one of the most aggressive types of brain tumors, is marked by its rapid proliferation and significant invasiveness [40]. Importantly, *NFE2L1* is implicated in glioma pathology. Research indicates that modulating *NFE2L1* expression can stimulate the Wnt signaling pathway, subsequently influencing glioma cell proliferation [41]. Our study further investigates the role of *NFE2L1* in gliomas by clarifying the intricate relationship between its expression and the polarization of TAMs within the glioma microenvironment, thereby underscoring the potential of *NFE2L1* as a viable therapeutic target for glioma treatment.

Our study found that *NFE2L1* is upregulated in glioma patients, highlighting its key role in malignancy progression [37, 42, 43, 44]. Intriguingly, our analysis revealed a positive correlation between *NFE2L1* expression levels and the infiltration of immune cells, notably macrophages. This association is particularly relevant in the context of recent studies that have begun to elucidate the multifaceted role of *NFE2L1* in modulating immune responses within the TME [8]. Our experiments showed *NFE2L1* promotes the M2 phenotype; its knockout decreased M2 markers (CD206 and CD163) and increased M1 markers (NOS2, CXCL10, CXCL9, and TNF‐α), indicating *NFE2L1*'s key role in macrophage polarization. Supporting our findings, prior research has established a link between *NFE2L1* and LPS‐induced macrophage activation. Notably, the deficiency of *NFE2L1* in macrophages was associated with an upregulation of M1 cytokines and a heightened response to LPS stimulation, facilitating the transition toward M1‐type macrophages [11]. The shift in macrophage polarization from M2 to M1 has the potential to bolster antitumor immunity [45], further underscoring the pivotal role of *NFE2L1* within the tumor immune microenvironment.

As a transcription factor regulating oxidative stress, the knockout of *NFE2L1* leads to significant downregulation of antioxidant‐related genes, such as glutathione synthesis‐associated enzymes—glutamate‐cysteine ligase catalytic subunit (GCLC) and heme oxygenase‐1 (HO‐1)—directly impairing the cell's ability to scavenge reactive oxygen species (ROS) and thus altering intracellular ROS levels [7, 46, 47]. Such elevated ROS levels promote pro‐inflammatory responses by oxidizing the mitochondrial DNA (mtDNA) or directly activating NLRP3, thereby enhancing caspase‐1 cleavage and maturation of IL‐1β and IL‐18 [48, 49]. This mechanism drives the polarization shift from M2 to M1 macrophages.

We demonstrated *NFE2L1*'s role in macrophage polarization affecting glioma cells, showing that *NFE2L1*‐deficient macrophages inhibit glioma cell migration, aligning with research on targeting macrophage polarization to stop tumor progression [50]. Conversely, in our previous experiments, the targeted knockout of *NFE2L1* in HepG2 cells enhanced malignant behavior of cells [51], indicating that *NFE2L1* plays different roles in different cell types and may have dual roles. We believe that this perspective is crucial for the future exploration of the functions of *NFE2L1*.

Furthermore, our in vivo experiments using *NFE2L1* ^*−/+*^ mice revealed that the loss of *NFE2L1* significantly reduced tumor growth and cell proliferation, supporting the notion that *NFE2L1* promotes gliomagenesis. This finding is corroborated by studies that identified *NFE2L1* as a key driver of tumor growth and a predictor of poor prognosis in various cancers [37, 39, 52, 53]. However, this contradicts our previous finding that *NFE2L1* promotes the malignant progression of liver cancer [30], a discovery that is worth considering. *NFE2L1* may regulate TAM polarization in tumorigenesis, with its role differing in liver cancer and glioma, acting as a double‐edged sword in cancer development and providing insights into its mechanisms in various pathologies. We analyzed samples from WT and *NFE2L1* ^*−*/+^ tumor model mice using mass spectrometry. Notably, in the *NFE2L1*‐deficient cohort, immune activation markers CD4 and CD3e were significantly upregulated. On one hand, this may result from *NFE2L1* deficiency leading to ROS accumulation, which could activate dendritic cells (DCs) or enhance tumor antigen release, thereby promoting the recruitment and activation of CD4^+^ T cells and CD8^+^ T cells (marked by CD3e) [54, 55]. On the other hand, *NFE2L1* deficiency may upregulate antigen‐presenting molecules, such as increased MHC‐I/II expression, thereby enhancing the helper function of CD4^+^ T cells. Given the complexity and heterogeneity of the TME, the mechanisms underlying this effect are likely multifaceted [56].

Moreover, the diminished expression of CD38 in the absence of *NFE2L1* points to a direct regulatory role of *NFE2L1* over this ectoenzyme, which is recognized for its ability to modulate immune responses. CD38 contributes to immune evasion by recruiting suppressive immune cells [19], thereby participating in cancer progression. These findings further underscore the potential of *NFE2L1* in sculpting the tumor immune microenvironment. However, *NFE2L1* shapes the tumor immune microenvironment, but its relationship with CD38 needs clarification, indicating potential for glioma therapy.

Anti‐PD‐1 therapy can effectively remodel the TME by altering TAM phenotypes, promoting the accumulation of M1‐type macrophages while suppressing M2‐type macrophage functions, thereby alleviating tumor‐mediated immunosuppression [57]. Concurrently, CD38 inhibitors improve T cell infiltration and function by reducing immunosuppressive factors in the TME. Specifically, CD38 inhibition effectively decreases the population of MDSCs and enhances T cell‐mediated antitumor responses, leading to improved immunologic status in the TME [58]. Furthermore, CD38 inhibitors can enhance T cell activation and proliferation by reducing levels of immunosuppressive cytokines such as IL‐10 and TGF‐β. This mechanistic insight provides a theoretical foundation for combination therapy, suggesting that the dual blockade of PD‐1 and CD38 may synergistically enhance immune cell function through multiple pathways, thereby improving therapeutic efficacy in glioma [59]. Thus, simultaneously targeting both PD‐1 and CD38 may disrupt the multi‐layered immunosuppressive network in the glioma microenvironment, offering a promising strategy for immunotherapy.

*NFE2L1* deficiency in tumors increases CD8 T cells and M1 macrophages, elevating CD4 and CD3e markers, while downregulating CD38 and PD‐L1. This mirrors past studies showing *NFE2L1*/MAFG promotes PD‐L1 via super enhancers and LPS, facilitating tumor immune escape [43]. The interaction between PD‐1 on T cells and PD‐L1 on tumor cells impedes the cancer immunity cycle [60], suggesting a model where inhibition of *NFE2L1* leads to immune activation and a shift of the TME toward a more inflammatory state.

In conclusion, our research reveals *NFE2L1*'s role in regulating TAMs in glioma, suggesting it as a therapeutic target. Suppressing *NFE2L1* may enhance immune activation and immunotherapy effectiveness. Future studies should explore *NFE2L1*'s molecular pathways and its inhibition's viability with immunotherapy. However, this study failed to elucidate the interaction between PD‐L1 and CD38, although some studies have suggested that CD38 may affect the expression of programmed death‐ligand 1 indirectly by regulating the intracellular signaling pathways [61]. Future research should aim to clarify the specific mode of interaction between programmed death‐ligand 1 and CD38 and their functional differences in different tumor types.

## Author Contributions

Qun Zhang contributed to formal analysis, data curation, conceptualization, investigation, methodology, and the writing and editing process of the review. Qiusi Tian was responsible for the collection of clinical data, acquisition of funding, and conducting animal experiments. Methodological contributions were made by Rongzhen Deng, Keli Liu, Shaofan Hu, Shiqun Chen, and Zhengwen Zhang. Hongzhao Lu provided equal supervision and participated equally in the writing and reviewing of the manuscript. Yiguo Zhang took the lead in project administration and oversaw the writing and review process.

## Conflicts of Interest

The authors declare no conflicts of interest.

## Supporting information


Appendix S1.

## Data Availability

The publication, along with the Supporting Information available online, contains all the necessary data required for assessing the conclusions drawn in the paper. Furthermore, any additional data pertinent to this study can be obtained by reaching out to the corresponding author, who can be contacted via the following email addresses: eaglezhang@fyust.org.cn or yiguozhang@cqu.edu.cn. For Figure 1A, the scRNA‐seq data were obtained from the SCEA dataset and the GEO dataset (GSE103224, GSE141982).
