# Supplementary material for: Inhibition of NFE2L1 Enables the Tumor‐Associated Macrophage Polarization and Enhances Anti‐PD1 Immunotherapy in Glioma
Source: CNS Neurosci Ther. 2025 Jul 17;31(7):e70488. doi: 10.1111/cns.70488 (PMC12271640; doi:10.1111/cns.70488)

Extended Data

Extended Data description

Extended Data Fig.1 NFE2L1$^{-/+}$ C57BL/6 mice were successfully constructed. (A) Schematic diagram of knockout site design. (B) Schematic diagram of knockout mice identification results.

Extended Data Fig.2 High expression of NFE2L1 is associated with immune invasion and low survival. (A) The expression of NFE2L1 was significantly correlated with the infiltration level of various types of immune cells of GBM and LGG. (B) The copy number of NFE2L1 was significantly correlated with the infiltration level of various types of immune cells of GBM and LGG. (C)Correlation analysis between high expression of NFE2L1 and TAM markers CD163 and CD68 based on CGGA database. (D) The correlation of NFE2L1, CD163 and CD68 expression level with survivals based on the CGGA database.

Extended Data Fig.3 The differentially expressed genes between wild-type TAM and NFE2L1$^{-/-}$ TAM groups through RNA-seq. (A) Principal component analysis (PCA) of RNA-seq data. (B) Volcano map visualization of differentially expressed genes. (C) GO enrichment analysis of differentially expressed genes. (D) KEGG enrichment analysis of differentially expressed genes.

Extended Data Fig.4 In macrophages knockout with NFE2L1, the expression of PDL1 and CD38 was downregulated.

Extended Data Fig.1

A

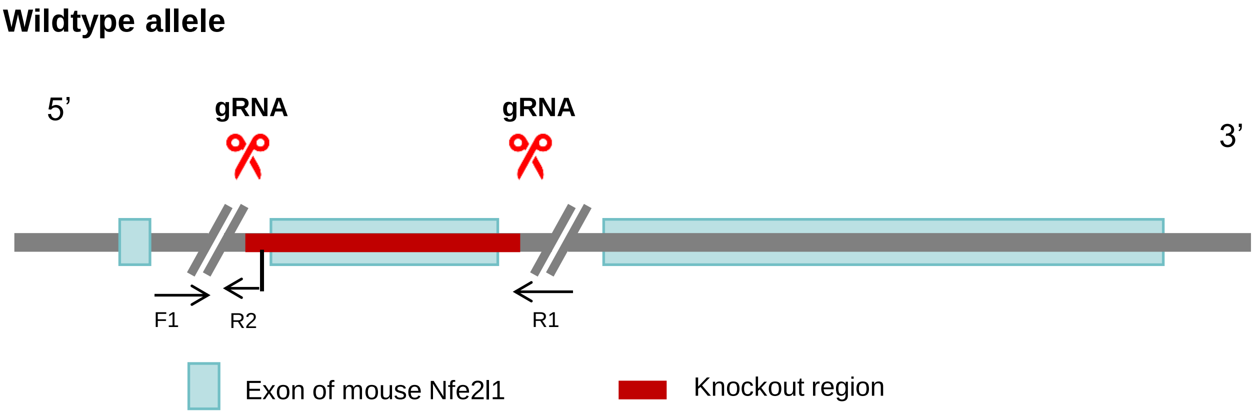

B

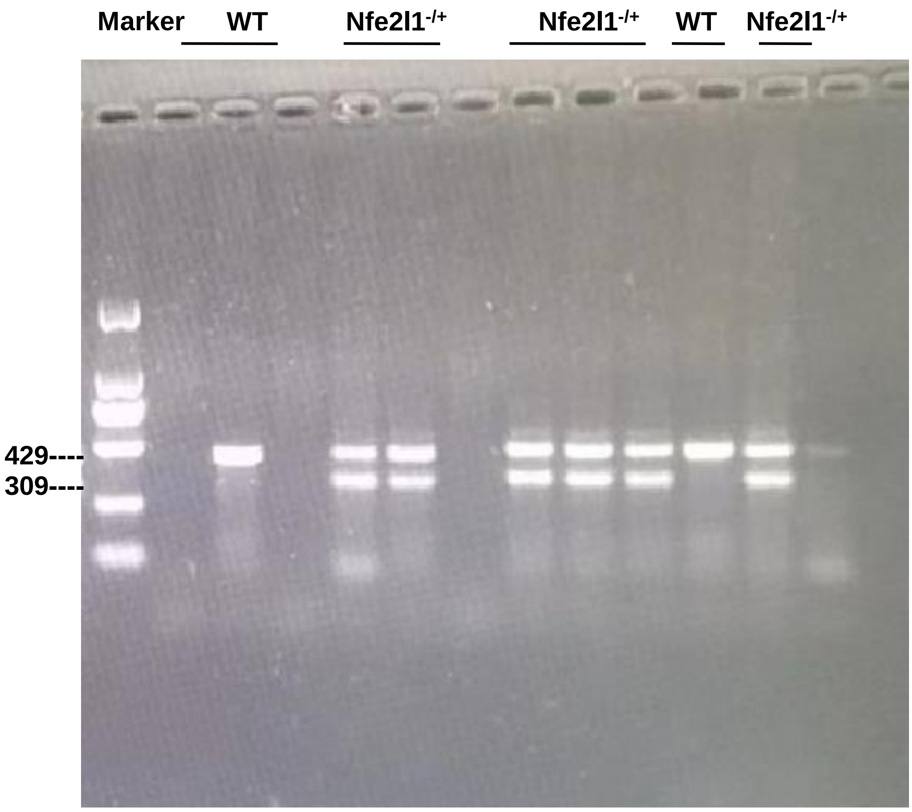

Extended Data Fig.2

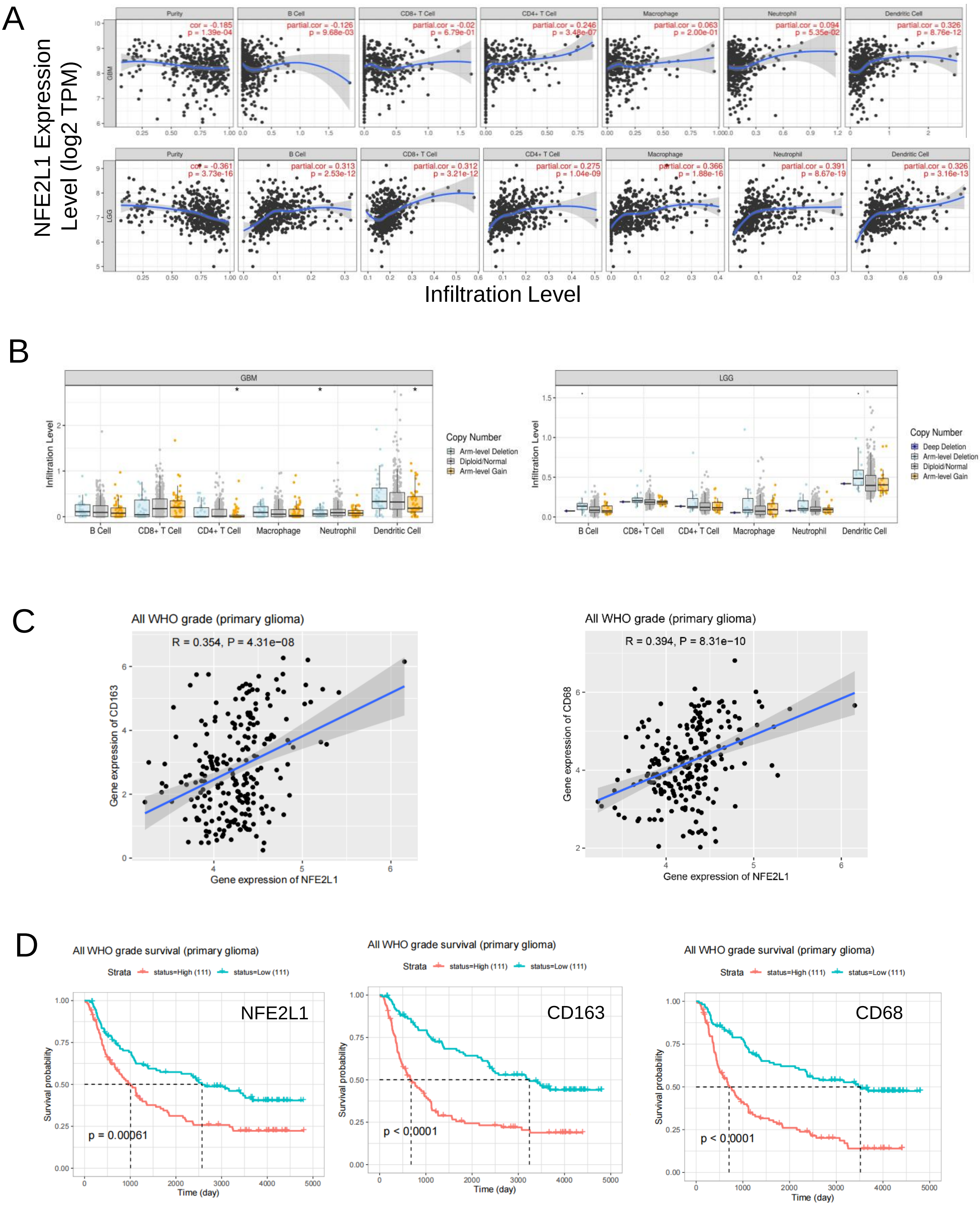

Extended Data Fig.3

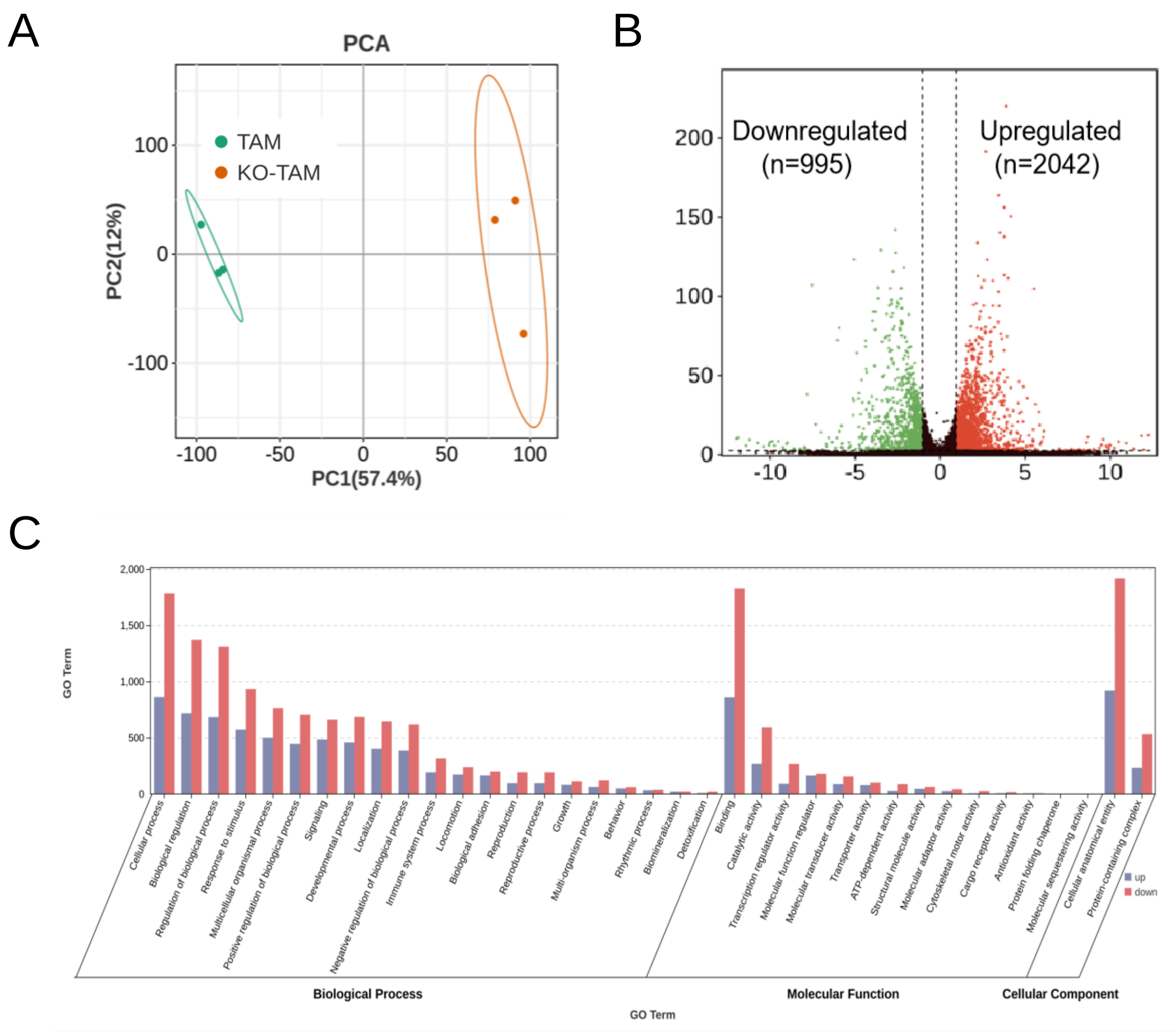

D

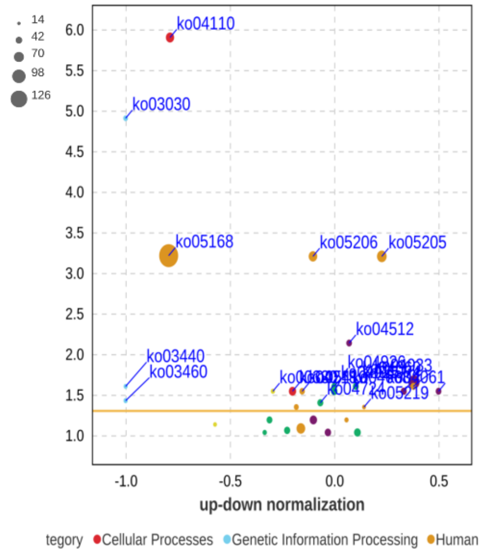

pathway top20

ID	Descrption	Number
ko04110	Cell cycle	47
ko03030	DNA replication	21
ko05168	Herpes simplex virus 1 infection	126
ko05206	MicroRNAs in cancer	49
ko05205	Proteoglycans in cancer	57
ko04512	ECM-receptor interaction	28
ko04060	Cytokine-cytokine receptor interaction	68
ko04933	AGE-RAGE signaling pathway in diabetic complications	29
ko04925	Aldosterone synthesis and secretion	28
ko04974	Protein digestion and absorption	29
ko03440	Homologous recombination	16
ko04061	Viral protein interaction with cytokine and cytokine receptor	28
ko05410	Hypertrophic cardiomyopathy	26
ko00330	Arginine and proline metabolism	17
ko04218	Cellular senescence	40
ko04926	Relaxin signaling pathway	34
ko04668	TNF signaling pathway	30
ko03460	Fanconi anemia pathway	17
ko04724	Glutamatergic synapse	30
ko05219	Bladder cancer	14

Extended Data Fig.4

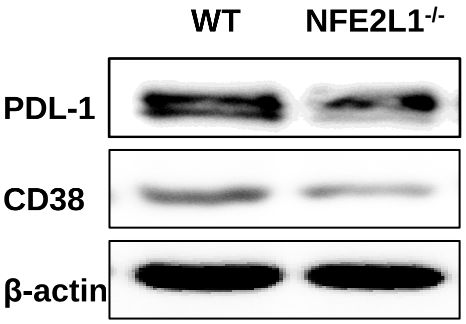

Supplement: Supplementary file 1 — Appendix S1. [file CNS-31-e70488-s001.zip › cns70488-sup-0001-AppendixS1.pdf]
