# Supplementary material for: Inhibition of NFE2L1 Enables the Tumor‐Associated Macrophage Polarization and Enhances Anti‐PD1 Immunotherapy in Glioma
Source: CNS Neurosci Ther. 2025 Jul 17;31(7):e70488. doi: 10.1111/cns.70488 (PMC12271640; doi:10.1111/cns.70488)
Supplement: Supplementary file 1 — Appendix S1. [file CNS-31-e70488-s001.zip › cns70488-sup-0002-AppendixS2.docx]

| 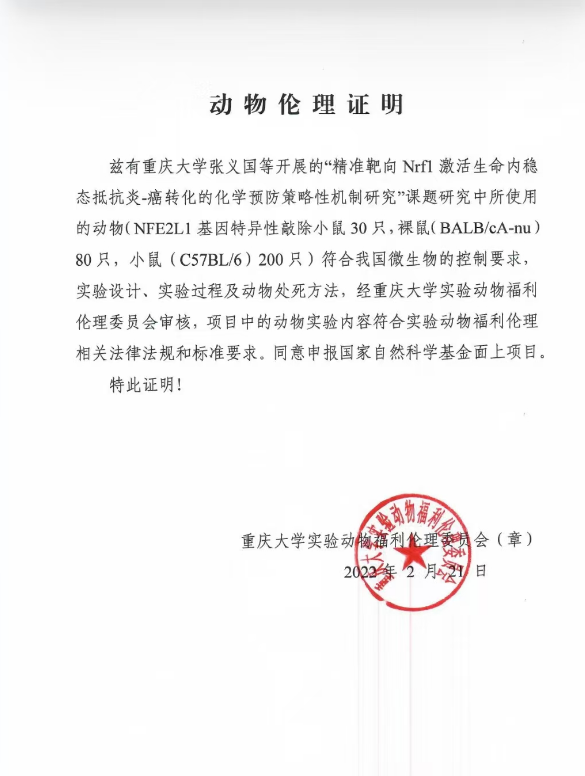 | Animal ethics proof  We have Chongqing university Zhang Yiguo such as "precise targeted Nrfl activate life steady resistance to inflammation-cancer transformation of chemical prevention strategy mechanism research" used in the research animals (NFE2L1 gene specific knockout mice, nude mice (BALB / cA-nu) 80, mice (C57BL/6) 200) conform to the requirements of microbial control experimental design, experimental process and animal death method, by Chongqing university experimental animal welfare ethics committee, the animal experiment content in the project meet the experimental animal welfare ethics related laws and regulations and standards. Agree to apply for the national Natural Science Foundation of China general project.  Hereby to certify!  Ethics Committee of Experimental Development and Release Welfare of Chongqing University (Chapter)  On February 21,2022 |
| --- | --- |
| 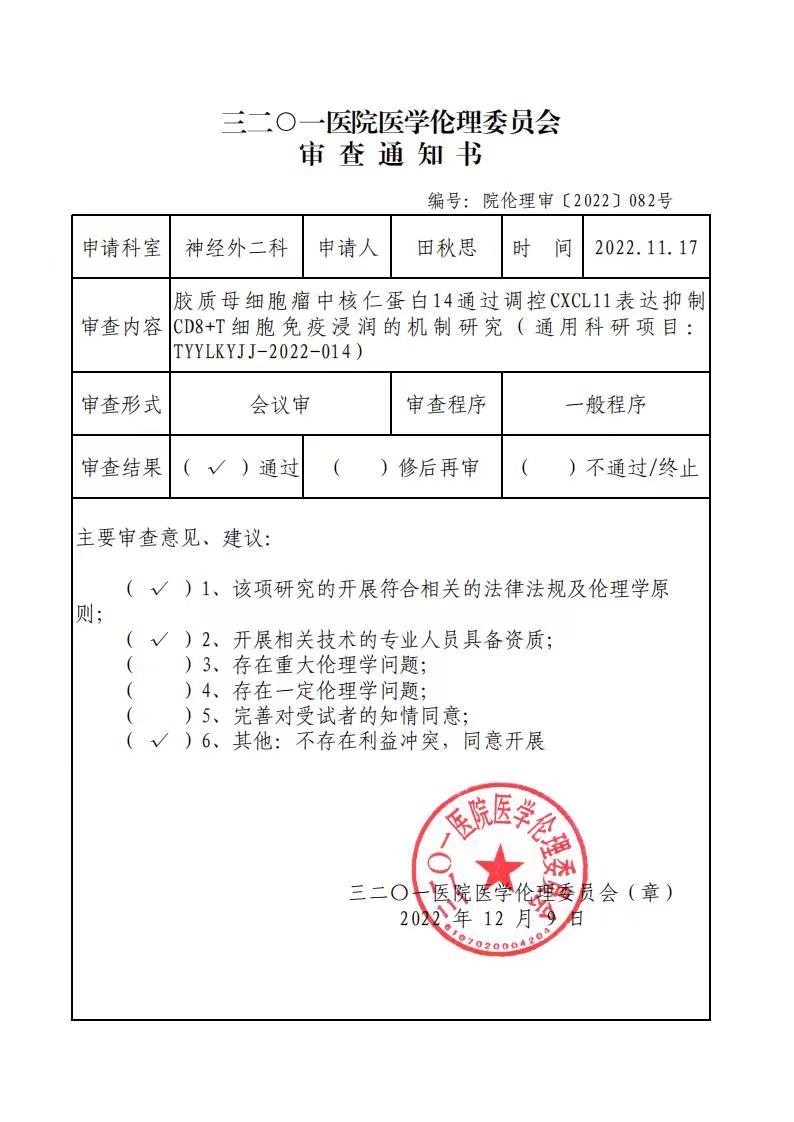 | Notice of review by the Medical Ethics Committee  of the 3201 Hospital  No.: Hospital ethics review [2022] 082   \| Apply for the department \| The second department of external nerve \| Proposer \| Qiusi Tian \| Time \| 2022.11.17 \| \| --- \| --- \| --- \| --- \| --- \| --- \| \| Review the content \| Study on the mechanism of nucleolar protein 14 inhibition of CD8 + T cells by regulating glial CXCL11 expression in glioblastoma (General research project: TYYLKYJJ-2022-014) \| \| \| \| \| \| Review form \| Meeting review \| Auditing routine \| General procedure \| \| \| \| Review the results \| ( √ ) pass through \| ( )Retrial after repair \| ( )Not passed / terminated \| \| \| \| Main review opinions and suggestions:  (√) 1. The study was conducted in accordance with the relevant laws and regulations and ethical principles;  (√) 2. Professionals carrying out relevant technology are qualified;  ( ) 3. Major ethical problems exist;  ( ) 4. There are certain ethical problems;  ( ) 5. Improve the informed consent of the subjects;  (√) 6, other: there is no conflict of interest, agree to carry out  3201 Hospital Medical Ethics Committee (Chapter)  December 9,2022 \| \| \| \| \| \| |
