# Supplementary material for: Inhibition of NFE2L1 Enables the Tumor‐Associated Macrophage Polarization and Enhances Anti‐PD1 Immunotherapy in Glioma
Source: CNS Neurosci Ther. 2025 Jul 17;31(7):e70488. doi: 10.1111/cns.70488 (PMC12271640; doi:10.1111/cns.70488)
Supplement: Supplementary file 1 — Appendix S1. [file CNS-31-e70488-s001.zip › cns70488-sup-0003-AppendixS3.docx]

**Supplementary Tables**

**Table S1. Clinical sample information.**

| No. | Age | Gender | Pathological diagnosis | WHO grade |
| --- | --- | --- | --- | --- |
| 1 | 55 | Male | Astrocytoma | 2 |
| 2 | 32 | Male | Oligoastrocytoma | 3 |
| 3 | 61 | Female | Glioblastoma | 4 |
| 4 | 70 | Female | Astrocytoma | 4 |
| 5 | 45 | Male | Glioblastoma | 4 |
| 6 | 53 | Female | Oligoastrocytoma | 3 |
| 7 | 67 | Female | Glioblastoma | 4 |
| 8 | 59 | Female | Glioblastoma | 4 |
| 9 | 72 | Male | Oligoastrocytoma | 3 |
| 10 | 57 | Male | Glioblastoma | 4 |

**Table S2. The primer pairs used for heterozygous recombinant mice.**

| **Name** | **Forward primers(5′-3′)** | **Reverse Primers (5′-3′)** |
| --- | --- | --- |
| **PCR Primers1** | **TGAGGCTCGCTTTTAAGGGT** | **TTAGCAAAGAATGGGAGCTTTGG** |
| **PCR Primers 2** | **TGAGGCTCGCTTTTAAGGGT** | **TGCCTTCCCATTTCTGATTTCTACT** |

Table S3. **The primer pairs used for RT-qPCR analysis.**

| **Name** | **Description** | **Forward primers(5′-3′)** | **Reverse Primers (5′-3′)** |
| --- | --- | --- | --- |
| **CD206** | **human** | **GGACGTGGCTGTGGATAAAT** | **ACCCAGAAGACGCATGTAAAG** |
| **CD163** | **human** | **TTTGTCAACTTGAGTCCCTTCAC** | **TCCCGCTACACTTGTTTTCAC** |
| **IL10** | **human** | **GACTTTAAGGGTTACCTGGGTTG** | **TCACATGCGCCTTGATGTCTG** |
| **ARG1** | **human** | **GTGGAAACTTGCATGGACAAC** | **AATCCTGGCACATCGGGAATC** |
| **NOS2** | **human** | **TTCAGTATCACAACCTCAGCAAG** | **TGGACCTGCAAGTTAAAATCCC** |
| **CXCL10** | **human** | **GTTTGAGACCAGCCTGACCAA** | **TCTCGGCTCACTGTAACCTC** |
| **CXCL9** | **human** | **CCAGTAGTGAGAAAGGGTCGC** | **AGGGCTTGGGGCAAATTGTT** |
| **TNF-α** | **human** | **GAGGCCAAGCCCTGGTATG** | **CGGGCCGATTGATCTCAGC** |
| **TGF-β1** | **human** | **CCACCTGCAAGACCATCGAC** | **CTGGCGAGCCTTAGTTTGGAC** |
| **NFE2L1** | **human** | **CATTCTGCTGAGTTTGATTGGGG** | **TTGTGGAACTGGGTCTGAGTAT** |
| **GAPDH** | **Human** | **CAACAGCCTCAAGATCATCAGC** | **AAGTGGTCGTTGAGGGCAATG** |

**Table S4. Main antibody information in the text.**

| Antibodies | Identifier | Source |
| --- | --- | --- |
| **NFE2L1** | **N/A** | **Zhang laboratory [1]** |
| **CD206（IF）** | **60143-1-IG** | **Proteintech Group** |
| **CD206（Flow cytometry）** | **APC-65155** | **Proteintech Group** |
| **CD206（IHC）** | **PA5-101657** | **Thermo Fisher** |
| **CD86** | **65165-1-IG** | **Proteintech Group** |
| **CD163** | **PE-65169** | **Proteintech Group** |
| **N-Cadherin** | **22018-1-AP** | **Proteintech Group** |
| **E-Cadherin** | **20874-1-AP** | **Proteintech Group** |
| **Snail2** | **12129-1-AP** | **Proteintech Group** |
| **Vimentin** | [**A19607**](https://abclonal.com.cn/catalog/A19607) | **ABclonal** |
| **CD8** | **ab184993** | **ABclonal** |
| **CD68** | **MA1-80133** | **Thermo Fisher** |
| **CD80** | **R381650** | **Zen-Bioscience** |
| **CD8** | [**A22219**](https://abclonal.com.cn/catalog/A22219) | **ABclonal** |
| **GSDMD** | [**A18281**](https://abclonal.com.cn/catalog/A18281) | **ABclonal** |
| [**Caspase**](https://abclonal.com.cn/catalog/A4888)**-1** | [**A20470**](https://abclonal.com.cn/catalog/A20470) | **ABclonal** |
| [**Caspase**](https://abclonal.com.cn/catalog/A4888)**-2** | [**A4888**](https://abclonal.com.cn/catalog/A4888) | **ABclonal** |
| **IL1B** | [**A22257**](https://abclonal.com.cn/catalog/A22257) | **ABclonal** |
| **Β-actin** | **AC038** | **ABclonal** |

References

[1] Y. Zhang, J.M. Lucocq, J.D. Hayes, The Nrf1 CNC/bZIP protein is a nuclear envelope-bound transcription factor that is activated by t-butyl hydroquinone but not by endoplasmic reticulum stressors, Biochem J, 418(2) (2009) 293-310, 10.1042/bj20081575.

Table S5. **Antibody information for mouse tissue samples**

| Antibody indicators | Large classification | notes |
| --- | --- | --- |
| CD45 | Leukocytes | Immune cells |
| CD3ε | Lymphoid cells | T lymphocytes |
| TCR β chain |  | α/β T cells |
| TCR γ/δ |  | γδ T cells |
| CD4 |  | CD4+T cells |
| CD8a |  | CD8+T cells |
| CD44 |  | Naïve/memory |
| CD62L |  | Naïve/memory |
| CD25(IL-2Rα) |  | Treg |
| CD127(IL-7Rα) |  | Treg/ILC |
| CD183(CXCR3) |  | Th1 |
| CD194(CCR4) |  | Th2 |
| CD196(CCR6) |  | Th17 |
| CD39 |  | Bystander |
| CD69 |  | activation |
| CD103(Integrin αE) |  | resident T/IEL/DC |
| CD278(ICOS) |  | checkpoint/ILC |
| CD279(PD-1) |  | exhaustion |
| CD19 |  | B cells |
| CD45R(B220) |  | B cells |
| IgD |  | Naïve B |
| CD27 |  | Memory B |
| CD161(NK-1.1) |  | NK cells |
| CD49b(pan-NK cells) |  | NK cells |
| CD11b | Myeloid cells | Myeloid cells, NK cells |
| CD11c |  | Antigen presenting cells |
| MHC II(I-A/I-E) |  | Antigen presenting cells |
| CD317(BST-2) |  | B cells, pDCs |
| CD117(c-kit) |  | ILC, mast cell |
| CD172a(SIRPα) |  | DC |
| Ly-6C |  | Monocytes |
| Ly-6G |  | MDSC/Neutrophils |
| CD64(FcγRI) |  | Macrophages |
| F4/80 |  | Macrophages |
| CD86 |  | M1-like |
| CD163 |  | M2-like |
| Siglec-F |  | Macrophages/DC/Eosinophils |
| CD192(CCR2) |  | Chemotaxis/cMono//macrophages and basophils |
| CX3CR1 |  | Chemotaxis/ncMono/microglia |
| CD38 |  | Macrophages, Lymphocytes, activation |
| CD274(PD-L1) |  | ligands of PD-1 |
