# Supplementary material for: Inhibition of NFE2L1 Enables the Tumor‐Associated Macrophage Polarization and Enhances Anti‐PD1 Immunotherapy in Glioma
Source: CNS Neurosci Ther. 2025 Jul 17;31(7):e70488. doi: 10.1111/cns.70488 (PMC12271640; doi:10.1111/cns.70488)

Fig.1 E-01 NFE2L1

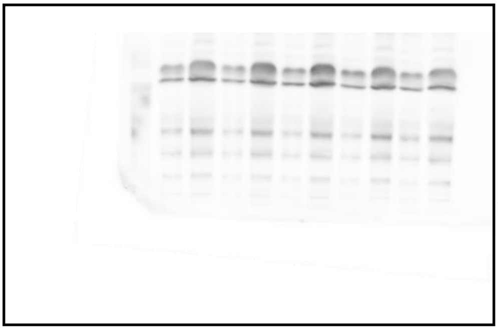

Fig.1 E-02 β-actin

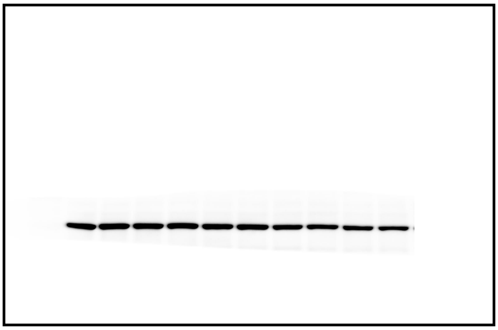

Fig.2 E-01 NFE2L1

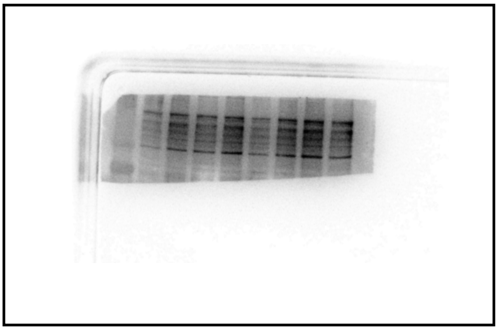

Fig.2 E-02 β-actin

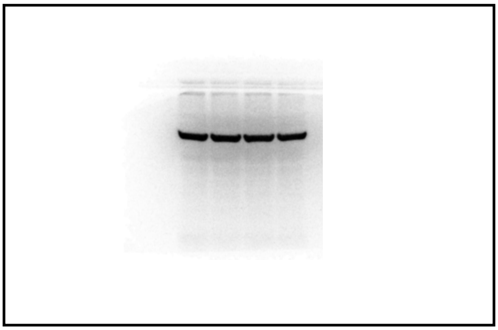

Fig.3 A-01 NFE2L1

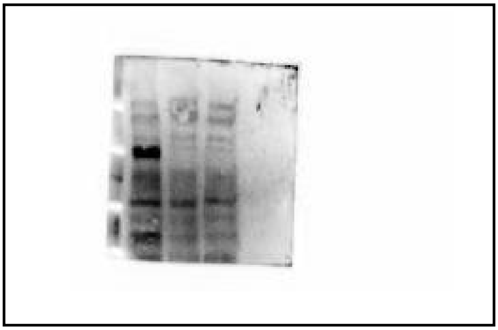

Fig.3 A-02 β-actin

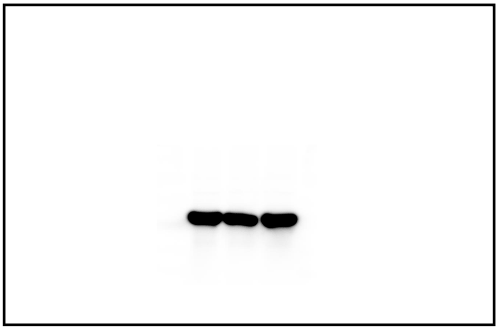

Fig.3 D-01 N-cad

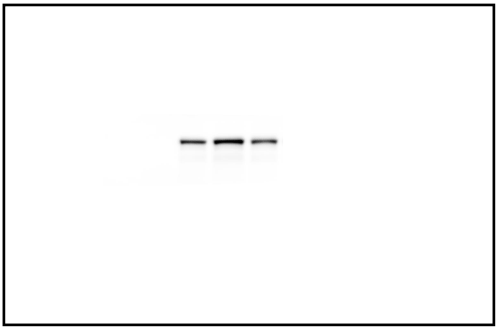

Fig.3 D-02 E-cad

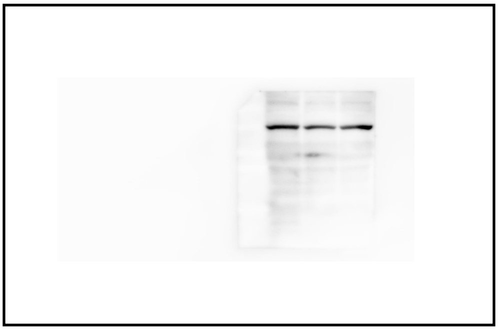

Fig.3 D-03 Vimentin

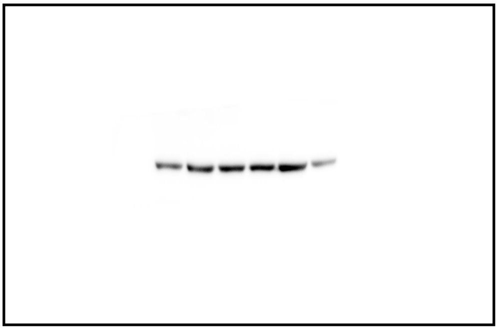

Fig.3 D-04 Snail

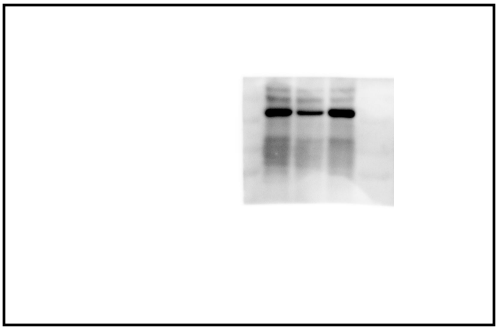

Fig.3 D-05 β-actin

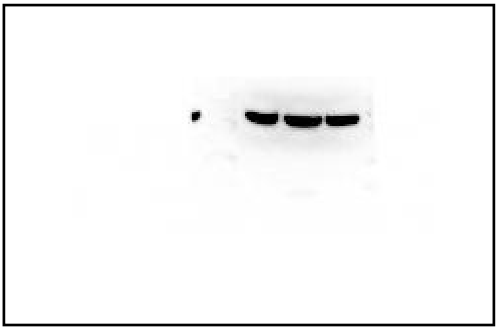

Fig.3 D-06 N-cad

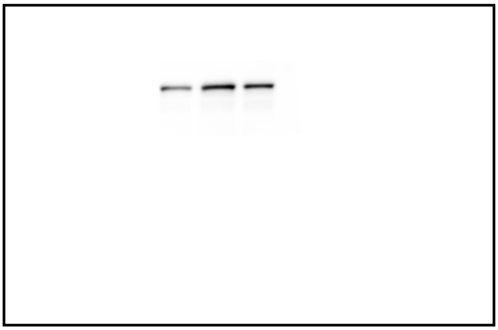

Fig.3 D-07 E-cad

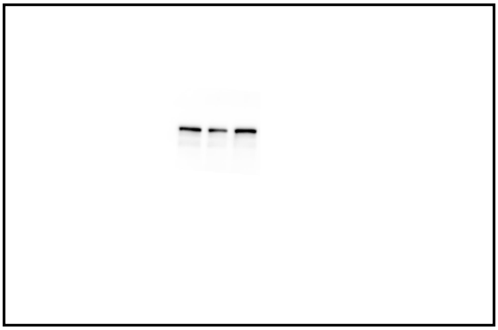

Fig.3 D-08 Vimentin

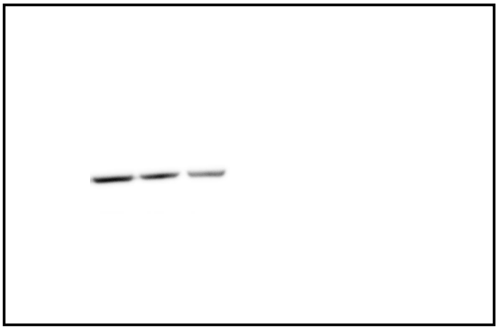

Fig.3 D-09 Snail

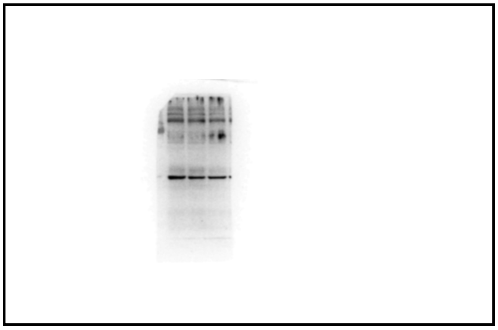

Fig.3 D-10 β-actin

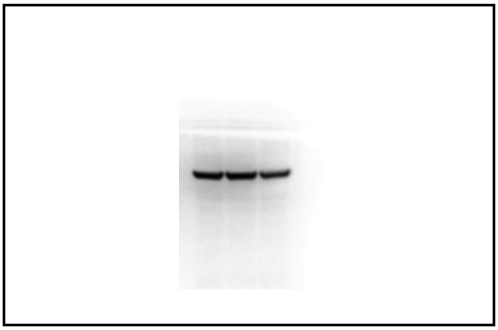

Supplement: Supplementary file 1 — Appendix S1. [file CNS-31-e70488-s001.zip › cns70488-sup-0004-AppendixS4.pdf]
